# Diagnostic performance of serum IgG4 level for IgG4-related disease: a meta-analysis

**DOI:** 10.1038/srep32035

**Published:** 2016-08-25

**Authors:** Wen-long Xu, Ying-chun Ling, Zhi-kai Wang, Fang Deng

**Affiliations:** 1 Department of Clinical Laboratory, Baoshan Branch of Shanghai Huashan Hospital, Baoshan Renhe Hospital, Shanghai 200431, China; 2 Department of Clinical Laboratory, Shaoxing Seventh People’s Hospital, Zhejiang Shaoxing 312000, China; 3 Department of Clinical Laboratory, Shanghai Family Planning Hospital, Shanghai 200032, China; 4 Department of Clinical Laboratory, Anhui Provincial Tumor Hospital, Anhui Hefei 230031, China

## Abstract

An elevated serum IgG4 level is one of the most useful factors in the diagnosis of IgG4-related disease (IgG4-RD). In this study, we performed a meta-analysis of the published articles assessing the diagnostic accuracy of serum IgG4 concentrations for IgG4-RD. The databases of MEDLINE/PubMed, EMBASE and Web of Science were systematically searched for relevant studies. Sensitivities and specificities of serum IgG4 in each study were calculated, and the hierarchical summary receiver operating characteristic (HSROC) model with a random effects model were employed to obtain the individual and pooled estimates of sensitivities and specificities. In total, twenty-three studies comprising 6048 patients with IgG4-RD were included in the meta-analysis. The pooled sensitivity was 85% with a 95% confidence interval (CI) of 78–90%; the pooled specificity was 93% with a 95% CI of 90–95%. The HSROC curve for quantitative serum IgG4 lies closer to the upper left corner of the plot, and the area under the curve (AUC) was 0.95 (95% CI 0.93, 0.97), which suggested a high diagnostic accuracy of serum IgG4 for the entity of IgG4-RD. Our study suggests that serum IgG4 has high sensitivity and specificity in the diagnosis of IgG4-RD.

Immunoglobulin (Ig) G4-related disease (IgG4-RD) is an entity of emerging immune-mediated diseases characterised by the infiltration of IgG4-bearing plasma cells, elevated serum IgG4 concentration, and systemic disorders present in nearly all organs with the exception of cerebral parenchyma^1^,^2^,^3^. The role of IgG4 in autoimmune diseases was first proposed for sclerosing or autoimmune pancreatitis (AIP) in 2001^4^. Since then, various organs in addition to the pancreas have been reported as involved in IgG4-related conditions^5^. Notably, many previously-recognised diseases, including AIP, Mikulicz’s disease (MD), chronic fibrosing sialadenitis, and other sclerosing disorders, are now known to belong to the clinical spectrum of IgG4-RD^1^.

The organ-specific diagnostic criteria for patients with IgG4-RD is likely impossible to establish due to variable organ involvements and clinical symptoms^6^. However, comprehensive criteria are important in clinical practice, especially in differential diagnosis from malignancies. Based on the common features of IgG4-RD, the current diagnostic criteria from Japan, Asian-Korea Consensus and Comprehensive Diagnostic Criteria generally include clinical symptoms (swelling or masses of single or multiple organs), histopathologic findings (extensive infiltration of IgG4^+^ plasma cells and fibrosis), haematological examination (concentrations of serum IgG4 >135 mg/dl), and imaging modalities (narrowing of ducts and/or enlargement of organs)^6^,^7^,^8^. An elevated serum IgG4 level is one of the most helpful of these criteria. Previous studies demonstrated that serum IgG4 concentrations significantly increased in patients with IgG4-RD^4^,^9^,^10^. The measurement of the serum IgG4 concentration is a useful tool in disease diagnosis, evaluating disease activity and predicting the responsiveness and clinical improvement of steroid and rituximab therapy in patients with IgG4-RD^11^,^12^.

However, many diseases other than IgG4-RD have higher levels of IgG4, and studies have suggested an unsatisfactory performance of serum IgG4 detection in the diagnosis of IgG4-RD with poor specificity and positive predictive value despite a high sensitivity and negative predictive value^2^,^13^,^14^. Additionally, IgG4-RD is a rare disorder with low incidence compared with other autoimmune disease, and the small sample sizes in previous studies focusing on the features of serum IgG4 level frequently led to a variable diagnostic accuracy of IgG4 in patients with IgG4-RD^6^,^9^,^15^. Notably, IgG4-RD is a systemic fibro-inflammatory disease that has a similar pathogenesis and cardinal features in affected organs; however, it was rarely analysed as an extensive entity but rather an individual disorder focused on a single organ, especially the pancreas and salivary or lacrimal glands^2^,^11^,^16^,^17^,^18^,^19^.

Up to now, a comprehensive overview of the accuracy and precision of the serum IgG4 concentration for the diagnosis of all IgG4-RD has not been performed. We aimed to establish the diagnostic performance of the serum IgG4 concentration for IgG4-RD involving the pancreas, bile duct, salivary gland, and lacrimal gland from non-IgG4-RD and/or healthy controls.

## Methods

### Search strategy

We searched the electronic databases of MEDLINE (via PubMed), EMBASE and the Web of Science from 2000 to September 2015 in accordance with the Preferred Reporting Items for Systematic reviews and Meta-Analyses (PRISMA) guidelines^20^. The systemic search was conducted combining the terms “serum”, “immunoglobulin g4” OR “igg4”, as well as the terms “sclerosing pancreatitis” OR “autoimmune chronic pancreatitis” OR “autoimmune pancreatitis” OR “cholangitis” OR “sclerosing cholangitis” OR “Küttner tumor” OR “sialadenitis” OR “sclerosing sialadenitis” OR “sclerosing dacryoadenitis” OR “Mikulicz’s disease” OR “igg4-rd” OR “igg4-related disease” with the species restriction of Human and language restriction of English. The relevant reference lists of the review articles were also screened to identify additional eligible articles not obtained in database searches.

### Data extraction and quality assessment

Prospective or retrospective case-control studies on the utility of serum IgG4 concentration in the diagnosis of IgG4-RD were deemed eligible for inclusion in the meta-analysis. The studies also met the criteria in that serum IgG4 concentration with an unambiguous cut-off value had been evaluated between IgG4-RD with a wide variety of organs involved and other diseases, as well as healthy controls. Articles with a larger sample size or more recently published articles were included when they used the same case series. Studies for which inadequate data for confirming the diagnosis of IgG4-RD and those assessing the role of IgG4 in the pathogenic mechanism were excluded. Conference or poster abstracts without sufficient clinical information or subsequent publication in full text were excluded. Studies with fewer than 10 included patients or based on animal or cell cultures were also excluded.

Risk of bias and applicability were critically assessed according to the revised Quality Assessment of Diagnostic Accuracy Studies (QUADAS-2) tool, which has 4 key domains including patient selection, index text, reference standard, and flow and timing^21^. Risk of bias and applicability concerns were judged as “low,” “high,” or “unclear”. Data extraction and quality assessment were performed by two reviewers (Wen-long Xu and Ying-chun Ling), and disagreements were resolved by discussion.

### Statistical analysis

The forest plot of individual and summarised sensitivity and specificity along with 95% confidence intervals (95% CIs) of the included studies were generated to graphically represent the diagnostic value of serum IgG4 in IgG4-RD. Subsequently, a hierarchical summary receiver operating characteristic (HSROC) model with a corresponding 95% confidence contour and 95% prediction contour was calculated. A bivariate random effects model following the DerSimonian-Laird method with a corresponding test of heterogeneity was used for data pooling. The heterogeneity across studies included in the meta-analysis was statistically detected using a Q test and I^2^ statistics, which ranged from 0 to 100% and were interpreted as representing low, medium and high inconsistency with the values of ≤25%, ≤50% and ≤75%, respectively, in accordance with the proposal of Higgins and Thompson^22^. Stratified analysis and meta-regression based on variations in features of ethnicity, spectrum of IgG4-RD and detection method were performed to explore potential sources of heterogeneity. Publication biases were tested using Egger precision weighted linear regression tests and sensitivity analysis and demonstrated graphically using funnel plots. The causes of heterogeneity were further assessed using a sensitivity analysis in which the sequential omission of individual studies was performed to analyse the influence of a single study on the overall detection rate of IgG4-RD. The meta-analysis of the data was conducted using the Stata/SE version 13.1 (StataCorp LP, Texas, USA). P < 0.05 was considered statistically significant.

## Results

### Study identification and selection

**Figure 1:**
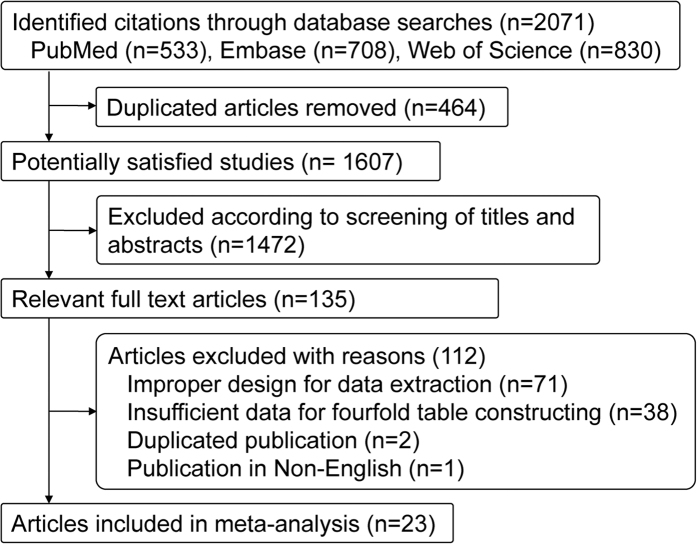
Study flow chart.

The initial keyword search yielded 2071 potentially relevant studies from the databases of PubMed (n = 533), Embase (n = 708), and the Web of Science (n = 830). After 464 duplicates were discarded, 1607 articles remained, of which the title and abstract were screened for eligibility in the meta-analysis. In accordance with the predefined inclusion criteria, 1472 articles were removed, and the remaining 135 articles were deemed potentially relevant. Following the further review of the full text articles, 111 studies were excluded due to improper design for data extraction (n = 70), insufficient data for fourfold table construction (n = 38), duplicated publication (n = 2) and published in a language other than English (n = 1). Finally, 23 articles were considered as eligible and included in this meta-analysis (Fig. 1). No disagreement occurred between the two reviewers.

### Study characteristics and methodological quality

In total, 6048 patients in 23 studies were included in the spectrum of IgG4-RD including AIP (13 studies), MD (3 studies), IgG4-associated cholangitis (6 studies) and IgG4-RD without further classification according to the organ involved (4 studies). The study type included 10 retrospective studies, 6 prospective studies and 7 studies without reporting the study type. Ethnicity included Asian in 15 studies with 3931 patients and Caucasian in 8 studies with 2117 patients (Table 1).

Overall, the included studies were of moderate methodological quality according to the QUADAS-2 tool. The high risk of bias and concerns of applicability regarding patient selection were introduced because all included studies were of a randomised or case-control design. The risk of bias for the index test and reference standard remained either (1) unclear because the index test and/or reference standard were interpreted double-blindly in all included studies with the exception of one^23^ or (2) high because the index test of serum IgG4 was not reported in five studies^24^,^25^,^26^,^27^,^28^. There were no major concerns regarding the applicability of the reference standard for the included studies. The risk of bias on flow and timing arose from the fact that the description of the interval and interventions between index tests and the reference standard were not reported in all studies (Table 1).

### Overall diagnostic accuracy

**Figure 2:**
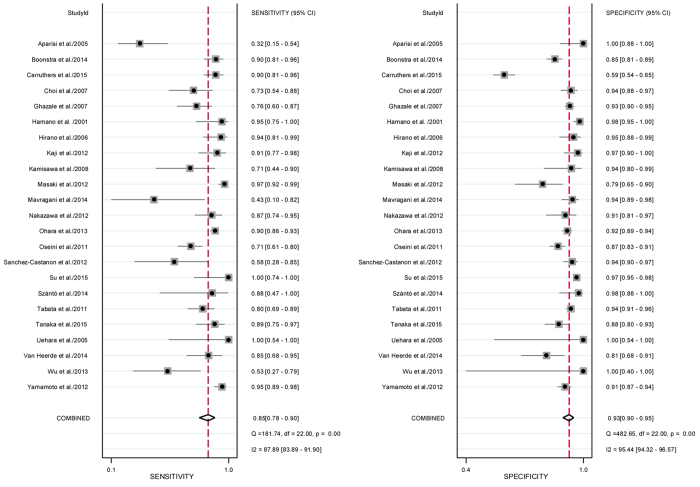
Forrest plot of the sensitivity and specificity of serum IgG4 demonstrating individual and summary sensitivity and specificity for the per-patient diagnosis of IgG4-RD. The corresponding heterogeneity of Q test with p < 0.01 and I^2^  > 75% suggests significant heterogeneity.

**Figure 3:**
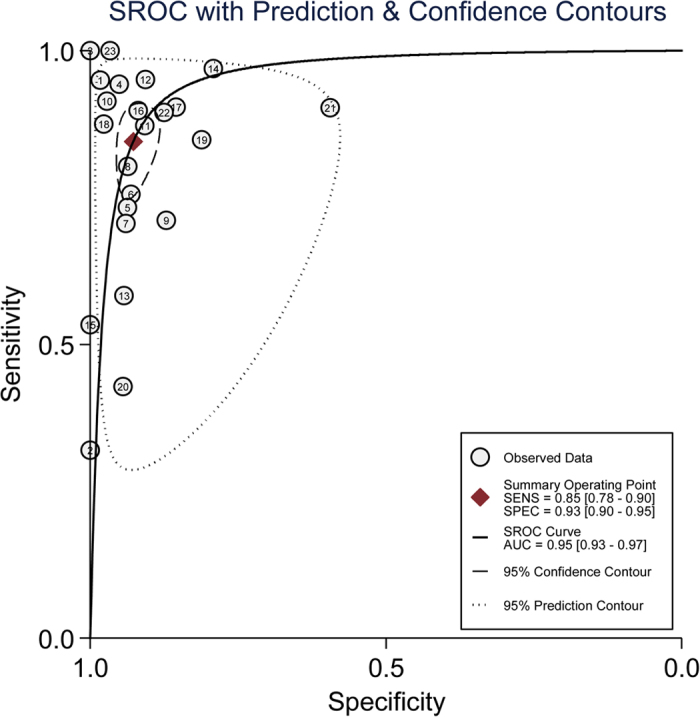
Hierarchical summary receiver operating characteristic (HSROC), with 95% confidence contour and 95% prediction contour, illustrating the summary operating point and study estimate of the sensibility and specificity for serum IgG4.

Serum IgG4 with a cut-off value ranging from 130 mg/dL to 200 mg/dL was detected using the assay methods of nephelometry (15 studies), single radial immunodiffusion (2 studies) and ELISA (1 study). Notably, the assay of serum IgG4 was not reported in 5 studies. The sensitivity and specificity of serum IgG4 concentration for the diagnosis of IgG4-RD ranged from 32% to 100% and from 59% to 100%, respectively (Table 2). The pooled sensitivity and specificity were 85% with a 95% confidence interval (CI) of 78–90% and 93% with a 95% CI of 90–95%, respectively (Fig. 2). The Q tests for pooled sensitivity and specificity were 181.74 with I^2^ of 87.89 (95% CI 83.89%, 91.90%) (p < 0.01) and 482.65 with I^2^ of 95.44 (95% CI 94.32%, 96.57%) (p < 0.01), both of which suggested significant heterogeneity (Fig. 3). The diagnostic odds ratio and positive and negative likelihood ratios were 70 (95% CI 42, 116), 11.6 (95% CI 8.1, 16.5) and 0.17 (95% CI 0.11, 0.24), respectively. These results strongly indicated that the higher rate of serum IgG4 positivity increased the chance of diagnosing IgG4-RD.

**Figure 4:**
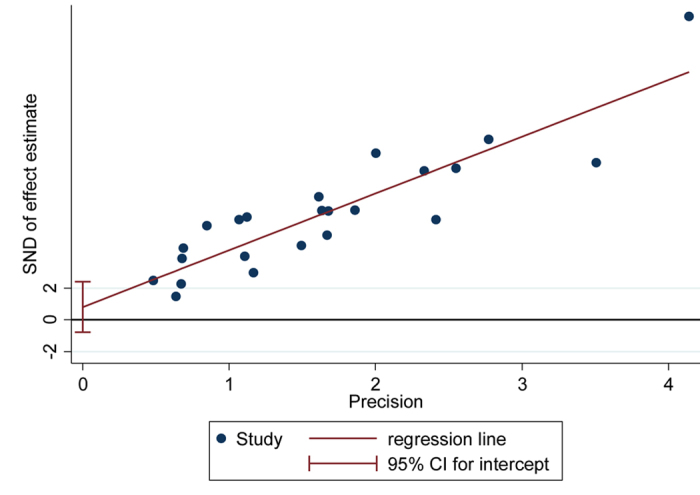
Egger tests for assessment of publication bias. (SND: standard normal deviate).

The diagnostic values of the studies were demonstrated in a HSROC graph in which the summary operating point represents the pooled sensitivity and specificity, as well as 95% confidence and the prediction region represents 95% CI of the pooled and individual sensitivity and specificity. The HSROC curve for quantitative serum IgG4 lies closer to the upper left corner of the HSROC plot, and the area under the curve (AUC) was 0.95 (95% CI 0.93, 0.97), which suggested an impressive diagnostic accuracy of serum IgG4 for the entity of IgG4-RD. Finally, the curve is symmetrical with the Z statistic of −0.58 (p = 0.564), which also indicates a high diagnostic accuracy for serum IgG4 (Fig. 4).

### Subgroup analysis and publication bias

The subgroup analysis was performed according to study period (published before vs. after 2011), study design (designed prospectively vs. retrospectively), sample size (less than 150 vs. more than 150), ethnicity (Asian vs. Caucasian), and serum IgG4 concentration detection assay (nephelometry vs. another method). The sensitivity of serum IgG4 was higher for (1) the studies published after 2011, (2) retrospective studies performed before 2011 that used detection methods other than assays, and (3) prospective studies using the method of nephelometry, whereas specificity was significantly different between the subgroups of all variables for the diagnosis of IgG4-RD (Table 3).

**Figure 5:**
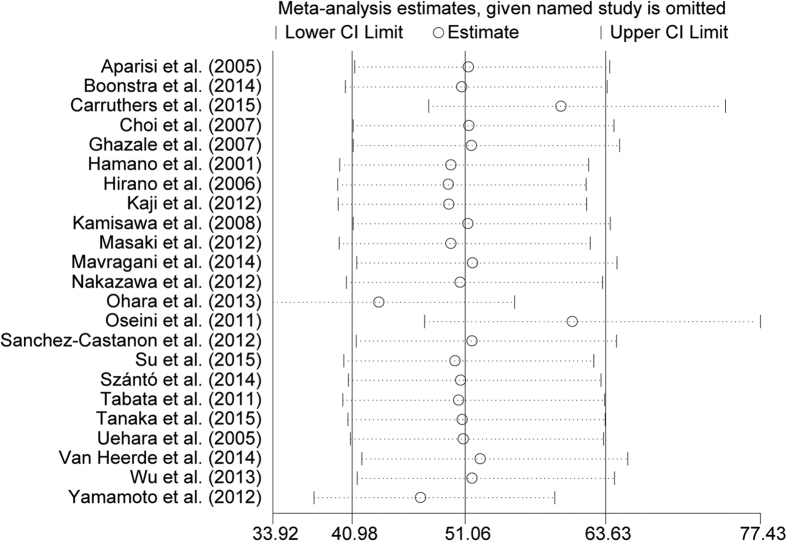
Meta-analysis estimates, given named study is omitted. The results shows that only little variation in summarized detection rate estimates of IgG4-RD is induced by omission of the one selected study. No systematic bias is identified although strong heterogeneity amongst studies is indicated. (Mantel-Haenzel fix model, statistic of relative risk).

Egger’s regression test did not reveal any publication bias arising from small-study effects (p  =  0.30) (Fig. 4). A sensitivity analysis suggested only a minor influence for diagnostic accuracy of omitting single-study estimates from 3 studies with larger sample sizes^2^,^29^,^30^; however, the estimates still fall within the indicated spread of lower and higher CI limits (Fig. 5).

## Discussion

Accurately differentiating IgG4-RD from malignancies such as pancreatic cancer is very important to avoid unnecessary surgeries. Compared with the histopathological criteria, the detection of serum IgG4 is one of the most convenient and valuable non-invasive examinations in clinical practice for the diagnosis of IgG4-RD, especially in disease screening at an early stage. However, because the positive rate of serum IgG4 varied according to different studies, the diagnostic accuracy of this method is controversial. A previous meta-analysis published in 2009 demonstrated that the serum IgG4 is a good marker of a single disease of AIP with a pooled sensitivity ranging from 82.3% to 89.3% and a specificity of 94.6% to 95.8% according to different control^31^. Beyond that, no studies were carried out to systematically summarise the currently available data on the performance of serum IgG4 for the diagnosis of IgG4-RD as an entity.

In the last decade, numerous studies have attempted to evaluate the diagnostic value of serum IgG4 in IgG4-RD. To summarise these studies, we conducted this meta-analysis, which included 23 studies comprising a total of 6048 patients with IgG4-RD diagnosed with different criteria. The key findings of our analysis are that serum IgG4 has a very high accuracy for sensitivity (85%) and specificity (93%) in detecting IgG4-RD involving the pancreas, bile duct, salivary gland, or lacrimal gland from non-IgG4-RD involving the same organs and/or healthy controls (or both). The serum IgG4 has a higher summarised sensitivity and specificity compared with the histopathological method using the infiltration of IgG4-positive plasma cells in which the pooled sensitivity and specificity were 58.8% and 90.2%, respectively^32^. The diagnostic value of serum IgG4 remained significant with a sensitivity range from 78% to 88% and a specificity range from 90% to 95% when analysed separately in different subgroups. An association between the sensitivity or specificity and the study period, study, sample size, ethnicity, or detection assay of serum IgG4 were also identified using meta-regression and subgroup analysis. The findings of such an assessment are useful both in providing evidence-based patient information in clinical application and further investigation.

Our meta-analysis had several limitations. Firstly, there was inevitable heterogeneity in the meta-analysis of diagnostic accuracy due to the variability in design characteristics and the poor quality of reporting in the primary studies. The quality of the studies included in the meta-analysis was modest for our meta-analysis because ten studies were retrospective designed^19^,^23^,^24^,^25^,^26^,^27^,^28^,^29^,^33^,^34^, six studies were prospective designed^11^,^16^,^18^,^30^,^35^,^36^, and seven studies did not report the study design^2^,^9^,^10^,^15^,^17^,^37^,^38^. The QUADAS-2 tools for the methodological assessment indicated that other contributors to the potential heterogeneity across the studies result from the risk bias in patient selection due to the diagnosis of IgG4-RD on the basis of multiple criteria, as well as due to the detection method for serum IgG4 despite the clear cut-off value given in several studies^24^,^25^,^26^,^27^,^28^. Regarding procedure and timing, the interval and whether there were any interventions between the index tests and the reference standard were not described in all studies. Secondly, because differing cutoff values were used in the same primary studies^2^,^11^,^18^,^35^, widely accepted values varying from 135 mg/dL (range 130 mg/dL to 200 mg/dL) were employed in the meta-analysis. It was not possible to assess a threshold effect for an optimum serum IgG4 concentration. Finally, several studies with high quality were excluded because the results were reported in the form of means and SD, which may contribute to the heterogeneity and may have impaired the stringency of the meta-analysis.

Serum IgG4 is a more cost-effective, easy and a time-efficient assay that can be carried out to detect IgG4-RD. We conclude that this meta-analysis has achieved its primary objectives by demonstrating that the detection of serum IgG4 has high sensitivity and represents a specific investigative modality in the detection of IgG4-RD as an indicator. However, this does not necessarily indicate that the positive or negative serum IgG4 could be used to rule out (or confirm) a diagnosis of IgG4-RD. A diagnosis based on IgG4-RD should be made after comprehensive analysis that considers clinical symptoms, as well as histopathologic, haematological, and imaging findings. The histopathological assessment of biopsy specimens from the involved tissues remains the cornerstone in both the definite diagnosis of IgG4-RD and the exclusion of malignancies. In summary, appropriate considerations and cautious interpretations of these findings combined with other parameters (especially pathological examination) are highly recommended, and additional studies that evaluate the accuracy of IgG4 in a wider spectrum of IgG-RD are needed.

## Additional Information

**How to cite this article**: Xu, W.- *et al*. Diagnostic performance of serum IgG4 level for IgG4-related disease: a meta-analysis. *Sci. Rep.* **6**, 32035; doi: 10.1038/srep32035 (2016).

## Figures and Tables

**Table 1:** Characteristics of included studies.

Author	Year	Study location	Ethnicity	Study design	Criteria for IgG4-RD	IgG4-RD disease (No.)	Control (No.)	Risk of bias*	Applicability concerns*
Aparisi *et al*.	2005	Spain	Caucasian	NR	Histopathological, clinical, and laboratory parameters	AIP (25)	ICP (29)	P^†^	No
Boonstra *et al*.	2014	Dutch	Caucasian	Prospective	Mayo Clinic’s HISORt criteria	IAC (73)	PSC (n = 310)	P	No
Carruthers *et al*.	2015	USA	Caucasian	NR	International pathology consensus guideline (2012)	IgG4-RD (72)	AID (n = 11), other disease (n = 397)	P	No
Choi *et al*.	2007	Korea	Asian	NR	Asan Medical Center of Korea	AIP (30)	PC (n = 76); CP (n = 67)	P	No
Ghazale *et al*.	2007	USA	Caucasian	Prospective	Mayo Clinic’s HISORt criteria	AIP (45)	PC (n = 135), other pancreatic diseases (n = 268); other disease (n = 62)	P	No
Hamano *et al*.	2001	Japan	Asian	Retrospective	Ultrasonographic, clinical, and laboratory performance	AIP (20)	PC (n = 70), CP (n = 45), PBC (n = 20), PSC (n = 8), SS (n = 11), HC (n = 20)	P	No
Hirano *et al*.	2006	Japan	Asian	Retrospective	Japan Pancreas Society	AIP (35)	CP (n = 24), PSC (n = 11), PC (n = 23), biliopancreatic cancer (n = 23)	P	No
Kaji *et al*.	2012	Japan	Asian	Prospective	AIP: Asian criteria (Japan–Korea) and ICDC	AIP (35)	PC (n = 17), CP (n = 24), PSC (n = 7), biliary cancer (n = 23)	P	No
Kamisawa *et al*.	2008	Japan	Asian	Retrospective	Radiological, serological histological examination	AIP (17)	PC (n = 33)	P, I^#^	I
Masaki *et al*.	2012	Japan	Asian	Retrospective	Pathological and clinical manifestations	IgG4-RD (132)	SS (n = 33), other non-AID (n = 15)	P, I	I
Mavragani *et al*.	2014	Greece	Caucasian	Retrospective	Comprehensive criteria	IgG4-RD (7)	SS (n = 126)*	P	No
Nakazawa *et al*.	2012	Japan	Asian	Retrospective	IgG4-SC: Japanese criteria 2006; AIP: HISORt criteria	IAC (47)	PC (n = 26), PSC (n = 21), CCA (n = 18)	P, I	I
Ohara *et al*.	2013	Japan	Asian	Retrospective	ICDC, revised Japanese criteria	IAC (344)	PC (n = 245), PSC (n = 110), CCA (n = 149)	P	No
Oseini *et al*.	2011	USA	Caucasian	Prospective	Mayo Clinic’s HISORt criteria	IAC (97)	CCA (n = 287)	P	No
Sanchez-Castanon *et al*.	2012	Spain	Caucasian	Retrospective	Mayo Clinic’s HISORt criteria	AIP (12)	CP (n = 23), ICP (n = 26), AP (n = 11), PC (n = 21), SS (n = 9), T1DM (n = 40), HC (n = 45)	P	No
Su *et al*.	2015	China	Asian	NR	Japan criteria	IgG4-RD (12)	AID (n = 127), other disease (n = 818)	P	No
Szántó *et al*.	2014	Japan	Asian	NR	Japanese Research Committee (2011)	AIP, MD (8)	SS (n = 43)	P	No
Tabata *et al*.	2011	Japan	Asian	Prospective	AIP: Asian criteria (Japan–Korea); MD: Clinical performance and exclusive criteria	AIP, MD (66)	Other pancreatobiliary or salivary gland diseases (n = 488)	P	No
Tanaka *et al*.	2015	Japan	Asian	Retrospective	Japanese Biliary Association (2012)	IAC (38)	PSC (n = 120)	P, I	I
Uehara *et al*.	2005	Japan	Asian	NR	Histopathological, clinical, and laboratory parameters	AIP-SC (6)	PSC (6)	P	P
Van Heerde *et al*.	2014	Dutch	Caucasian	Prospective	ICDC, Asian or HISORT criteria, or comprehensive criteria	AIP (33)	PC (n = 53)	P	P
Wu *et al*.	2013	China	Asian	Retrospective	Pathological and radiologic manifestations	AIP (15)	Non-AIP (n = 4)	P, I	No
Yamamoto *et al*.	2012	Japan	Asian	NR	Mayo Clinic’s HISORt criteria; Japan criteria	AIP, MD, CFSA, DA (102)	AID (n = 206), other disease (n = 72), HC (n = 21)	P	P

IgG4-RD: IgG4-related disease; AIP: autoimmune chronic pancreatitis; MD: Mikulicz’s disease; IAC: IgG4-associated cholangitis; CFSA: Chronic fibrosing sialoadenitis; DA: IgG4-related dacryoadenitis; PC: pancreatic cancer; CP: chronic pancreatitis other than AIP; ICP: idiopathic chronic pancreatitis; PBC: primary biliary cirrhosis; SS: Sjögren’s syndrome; CCA: cholangiocarcinoma; HC: healthy control; T1DM: type 1 diabetes mellitus; AID: autoimmune diseases; ICDC: International consensus diagnostic criteria; NR: not reported. *QUADAS score; ^†^P: patient selection; ^#^I: index test.

**Table 2:** Diagnostic accuracy of serum IgG4 concentration for individual study.

Author	Year	Assay for IgG4	Cut-off (mg/dL)	Participant, n	True positive	False positive	False negative	True negative	Sensitivity	Specificity
Aparisi *et al*.	2005	Nephelometry	130	54	8	0	17	29	32%	100%
Boonstra *et al*.	2014	Nephelometry	140	383	66	45	7	265	90%	85%
Carruthers *et al*.	2015	Nephelometry	135	380	65	125	7	183	90%	59%
Choi *et al*.	2007	SRI	135	173	22	9	8	134	73%	94%
Ghazale *et al*.	2007	Nephelometry	140	510	34	32	11	433	76%	93%
Hamano *et al*.	2001	SRI	135	194	19	3	1	171	95%	98%
Hirano *et al*.	2006	Nephelometry	135	116	33	4	2	77	94%	95%
Kaji *et al*.	2012	Nephelometry	135	106	32	2	3	69	91%	97%
Kamisawa *et al*.	2008	NR	135	50	12	2	5	31	71%	94%
Masaki *et al*.	2012	NR	135	180	128	10	4	38	97%	79%
Mavragani *et al*.	2014	Nephelometry	135	133	3	7	4	119	43%	94%
Nakazawa *et al*.	2012	NR	135	112	41	6	6	59	87%	91%
Ohara *et al*.	2013	Nephelometry	135	848	309	41	35	463	90%	92%
Oseini *et al*.	2011	Nephelometry	140	384	69	37	28	250	71%	87%
Sanchez-Castanon *et al*.	2012	Nephelometry	130	187	7	10	5	165	58%	94%
Su *et al*.	2015	ELISA	135	957	12	32	0	913	100%	97%
Szántó *et al*.	2014	Nephelometry	135	51	7	1	1	42	88%	98%
Tabata *et al*.	2011	Nephelometry	135	554	53	31	13	457	80%	94%
Tanaka *et al*.	2015	NR	135	158	34	15	4	105	89%	88%
Uehara *et al*.	2005	Nephelometry	135	12	6	0	0	6	100%	100%
Van Heerde *et al*.	2014	Nephelometry	140	86	28	10	5	43	85%	81%
Wu *et al*.	2013	NR	200	19	8	0	7	4	53%	100%
Yamamoto *et al*.	2012	Nephelometry	144	401	97	28	5	271	95%	91%

**SRI:** single radial immunodiffusion; **NR:** not reported.

**Table 3:** Subgroup analysis of the sensitivity and specificity of serum IgG4 for the diagnostic performance of IgG4-RD.

Parameter	Category	No. of study	Sensitivity	p	Specificity	p
Period	Before 2011	9	78% (67–90%)	0.00	95% (92–98%)	0.00
	After 2011	14	88% (82–94%)		91% (87–95%)	
Study design	Prospective	6	83% (74–93%)	0.04	90% (86–94%)	0.00
	Retrospective	10	85% (77–92%)		93% (91–96%)	
Sample size	Less than 150	13	88% (82–94%)	0.35	91% (87–95%)	0.00
	More than 150	10	79% (67–90%)		95% (92–98%)	
Ethnicity	Asian	15	88% (83–93%)	0.63	94% (92–97%)	0.00
	Caucasian	8	73% (61–86%)		90% (84–95%)	
Assay	Nephelometry	15	83% (75–91%)	0.02	92% (89–95%)	0.00
	Other method	8	87% (78–96%)		94% (90–98%)	
